# Supplementary material for: Conditional chemoconnectomics (cCCTomics) as a strategy for efficient and conditional targeting of chemical transmission
Source: eLife. 2024 Apr 30;12:RP91927. doi: 10.7554/eLife.91927 (PMC11060718; doi:10.7554/eLife.91927)
Supplement: Figure 3—source data 3. [file elife-91927-fig3-data3.zip › Figure 3 source data3 part 2.zip!/Figure 3-Source data 3-part2/1060f2-1-CRISPResso_Report_gl5umwyq.zip!/CRISPResso_on_1060f2_1/1a.Read_barplot.pdf]

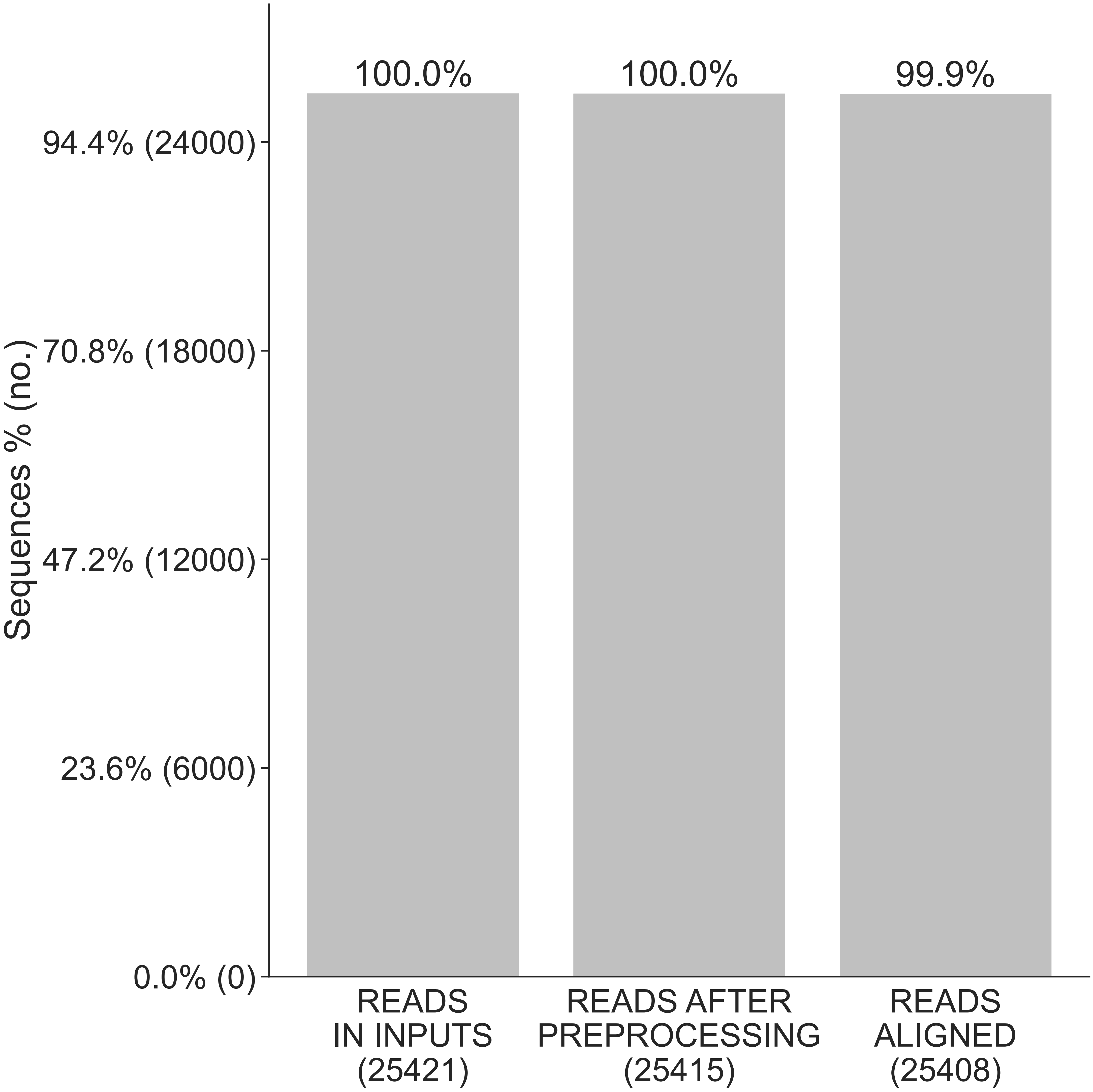

100.0%
100.0%
99.9%
94.4% (24000)
70.8% (18000)
47.2% (12000)
23.6% (6000)
0.0% (0)
Sequences % (no.)
READS IN INPUTS (25421)
READS AFTER PREPROCESSING (25415)
READS ALIGNED (25408)
